# Phase segregation due to ion migration in all-inorganic mixed-halide perovskite nanocrystals

**DOI:** 10.1038/s41467-019-09047-7

**Published:** 2019-03-06

**Authors:** Huichao Zhang, Xu Fu, Ying Tang, Hua Wang, Chunfeng Zhang, William W. Yu, Xiaoyong Wang, Yu Zhang, Min Xiao

**Affiliations:** 1 National Laboratory of Solid State Microstructures, School of Physics, and Collaborative Innovation Center of Advanced Microstructures, Nanjing University, Nanjing, 210093 China; 2 College of Electronics and Information, Hangzhou Dianzi University, Xiasha Campus, Hangzhou, 310018 China; 3 State Key Laboratory of Integrated Optoelectronics and College of Electronic Science and Engineering, Jilin University, Changchun, 130012 China; 4 Department of Physics, University of Arkansas, Fayetteville, AR 72701 USA

## Abstract

Semiconductor mixed-halide perovskites featured with a tunable energy bandgap are ideal candidates for light absorbers in tandem solar cells as well as fluorescent materials in light-emitting diodes and nanoscale lasers. These device advancements are currently hindered by the light-induced phase segregation effect, whereby ion migration would yield smaller-bandgap domains with red-shifted photoluminescence. Here we show that upon laser excitation all-inorganic mixed-halide nanocrystals unexpectedly exhibit a blue shift in the photoluminescence peak that can revert back in the dark, thus depicting the processes of ion migration out of and back to the originally excited nanocrystals. Interestingly, this reversible photoluminescence shift can also be induced by electrical biasing of mixed-halide nanocrystals without the injection of charge carriers. The above findings suggest that it is the local electric field that breaks the ionic bonds in mixed-halide nanocrystals, which could be a universal origin for light-induced phase segregation observed in other mixed-halide perovskite materials.

## Abstract (web summary)

Mixed-halide perovskites possess excellent semiconductor properties but suffer severely from notorious light-induced phase segregation effect. Here Zhang et al. employ simple photoluminescence measurements to link the effect to the local electric field induced ion migration process.

## Introduction

Semiconductor perovskites of methylammonium (MA) lead halide (MAPbX_3_, X = Br, Cl, I or mixtures thereof) have been drawing great research attention due to their superior performance in solar cells with a rapidly rising photo-conversion efficiency toward that of the crystalline silicon counterparts^1,2^. The main obstacle for commercial advancement of these organolead halide solar cells lies in the instability and degradation of light-harvesting MAPbX_3_ films^3^. Especially bothersome is the ion migration effect, which is believed to be responsible for the slow conductivity response^4^, anomalous current–voltage hysteresis^5,6^, giant dielectric constant^7^, and switchable photocurrent^8^ in the MAPbI_3_ perovskites. Meanwhile, ion migration is manifested in mixed-halide MAPbBr_*x*_I_3-*x*_ (0 < *x* < 3) perovskites as a light-induced segregation of iodine- and bromine-rich domains that can revert back to the original phase in the dark^9–12^. The iodine-rich domains in the MAPbBr_*x*_I_3-*x*_ perovskites would cause a red shift in the photoluminescence (PL) that has been similarly observed in the CsPbBr_*x*_I_3-*x*_ perovskites^13–16^, confirming a universal role played by the migration of halide ions in the light-induced phase segregation process. In addition to the bulk mixed-halide perovskites mentioned above, the phase segregation effect has also been observed in the CsPbBr_*x*_I_3-*x*_ nanocrystals (NCs) albeit from the electroluminescence measurements^17,18^. Given the nanoscale size of the iodine-rich domains^11,19^ that are preferentially located along grain boundaries of the bulk mixed-halide perovskites^11,16,20,21^, the CsPbBr_*x*_I_3-*x*_ NCs with a normally defective surface could provide a succinct understanding of the phase segregation process from a bottom-up point of view.

Here we focus on mixed-halide CsPbBr_1.2_I_1.8_ NCs to investigate the phase segregation effect at both the ensemble-film and the single-particle levels. With laser excitation, the ensemble film of CsPbBr_1.2_I_1.8_ NCs demonstrates a blue shift from about 630 to 520 nm in the PL peak that can revert back in the dark, which can be attributed to the migration of iodide ions out of and back to the originally excited NCs. For an isolated single CsPbBr_1.2_I_1.8_ NC, the PL is also shifted to the blue side upon laser excitation but never returns back in the dark, signifying the necessary existence of nearby NCs to channel the migration of iodide ions. Interestingly, the blue-shifted PL can also be induced when the CsPbBr_1.2_I_1.8_ NCs are electrically biased in the dark without the injection of excited-state charge carriers. This strongly suggests that it is the local electric field to break the iodide bonds that triggers the ion migration process in photo-excited CsPbBr_1.2_I_1.8_ NCs. While the enlarged energy bandgap is mainly caused by bromine enrichment in mixed-halide CsPbBr_1.2_I_1.8_ NCs, we additionally show that lattice distortion by the migration of iodide ions could also make a minor contribution, as verified by the observation of a blue shift in the PL peak as large as 20 nm from the single-halide CsPbI_3_ NCs upon laser excitation.

## Results

### Chemical synthesis and experimental conditions

According to a previously reported procedure^22^ (see Methods), mixed-halide CsPbBr_1.2_I_1.8_ NCs were similarly synthesized with a cubic shape and a side length of about 23 nm as estimated from the transmission electron microscopy (TEM) measurements (Supplementary Figure 1). From room-temperature solution measurements, the absorption and emission peaks of the CsPbBr_1.2_I_1.8_ NCs are located at 617 and 632 nm, respectively (Supplementary Figure 2). One drop of the concentrated or diluted solution of the CsPbBr_1.2_I_1.8_ NCs was spin-coated onto a fused silica substrate for the room-temperature optical characterizations of an ensemble film or single particles with a 405 nm picosecond laser operated at a repetition rate of 5 MHz (see Methods). The laser beam was focused onto the sample substrate with a spot size of about 500 nm so that different positions of the ensemble film or the single particles could be studied with a high spatial resolution.

### Optical measurements of high-density CsPbBr_1.2_I_1.8_ NCs

**Fig. 1:**
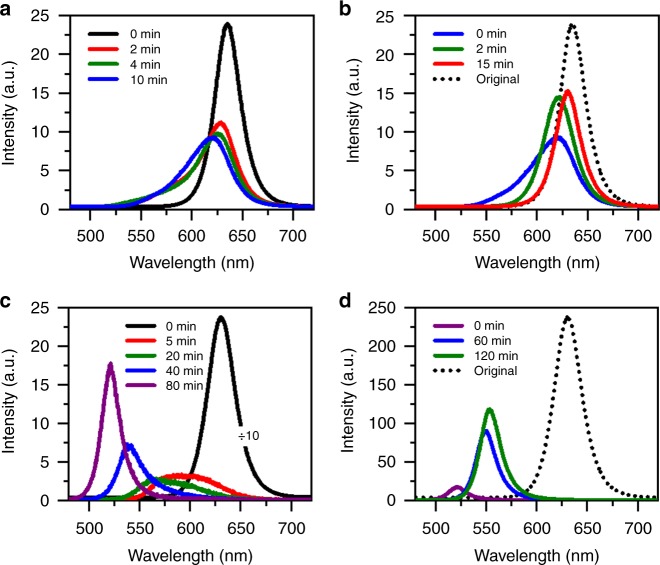
Light-induced phase segregation in ensemble film of CsPbBr_1.2_I_1.8_ nanocrystals (NCs). **a** Photoluminescence (PL) spectra measured at 0, 2, 4, and 10 min for one position of the ensemble film continuously excited at a laser power density of 30 W cm^−2^. The PL spectrum at each time point was acquired with an integration time of 1 s. **b** PL spectra measured for the same position of the ensemble film after the excitation laser had then been blocked for 0, 2, and 15 min. At each time point, the laser beam was unblocked for 1 s to acquire the PL spectrum still at 30 W cm^−2^. The original PL spectrum measured at the beginning of the laser excitation in **a** is also plotted. **c** PL spectra measured at 0, 5, 20, 40, and 80 min for another position of the ensemble film continuously excited at a laser power density of 15 kW cm^−2^. The PL spectrum at each time point was acquired with an integration time of 1 s. The PL spectrum measured at 0 min has been scaled down by 10 times for a good comparison. **d** PL spectra measured for this position of the ensemble film after the excitation laser had then been blocked for 0, 60, and 120 min. At each of the above time points, the laser beam was unblocked for 1 s to acquire the PL spectrum still at 15 kW cm^−2^. The original PL spectrum measured at the beginning of the laser excitation in **c** is also plotted

As shown in Fig. 1a for one position of the ensemble film of CsPbBr_1.2_I_1.8_ NCs, the PL peak was blue-shifted from the initial 635 nm toward a stable value of 618 nm after 10 min of continuous laser excitation at the power density of 30 W cm^−2^ (in comparison, the power density of 1 sun illumination is 100 mW cm^−2^). The reversibility of the above process is demonstrated in Fig. 1b, where the same position of the ensemble film showed a red shift in the PL peak that approached 631 nm after 15 min of being left in the dark. The above PL shifting process is strongly dependent on the laser excitation power, as can be seen in Fig. 1c for another position of the ensemble film excited at 15 kW cm^−2^. Within 80 min of laser excitation, the PL peak was blue-shifted all the way toward about 520 nm to coincide with optical emission from the single-halide CsPbBr_3_ NCs^18,23^_,_ while similar blue-shifted behavior was also observed in the corresponding absorption spectrum (Supplementary Figure 3). When this position of the ensemble film was then measured in the dark (Fig. 1d), the PL peak was also red-shifted but stopped only at 553 nm after 120 min to demonstrate a worse reversibility than that obtained at 30 W cm^−2^. Accompanying time-dependent shift of the PL peak, PL intensity of the ensemble film was also partially reversible, dropping by about 45% (90%) immediately after laser excitation at 30 W cm^−2^ (15 kW cm^−2^) and recovering in the dark to about 65% (45%) of the original value.

For the bulk films of mixed-halide perovskites^10,12^, light-induced phase segregation is universally causing a red shift in the PL due to exciton trapping into the iodine-rich regions with a smaller energy bandgap than that of the bromine-rich regions^9,15,24^. However, laser excitation here on the ensemble film of CsPbBr_1.2_I_1.8_ NCs reproducibly yielded a blue shift in the PL, suggesting that they were being transformed to the bromine-rich optical emitters owing to the preferred bond breaking and the subsequent escape of iodide ions. This can be largely understood since it has been documented both theoretically and experimentally that the Pb-Br bond is shorter and stronger than the Pb-I bond^24–29^, so that a higher binding energy would be associated with the ionization of the bromide ions^30^. In the bulk films of mixed-halide MAPbBr_*x*_I_3-*x*_ and CsPbBr_*x*_I_3-*x*_ perovskites, the light-induced iodine-rich domains are normally located at the grain boundaries^11,16,20,21^ that are full of impurities, vacancies or dangling bonds^31^. These grain boundaries can be well mimicked by the defective surfaces of CsPbBr_1.2_I_1.8_ NCs, and they can be envisioned to serve as a stopping site into which the light-freed iodide ions can diffuse.

### Optical measurements of low-density and single CsPbBr_1.2_I_1.8_ NCs

**Fig. 2:**
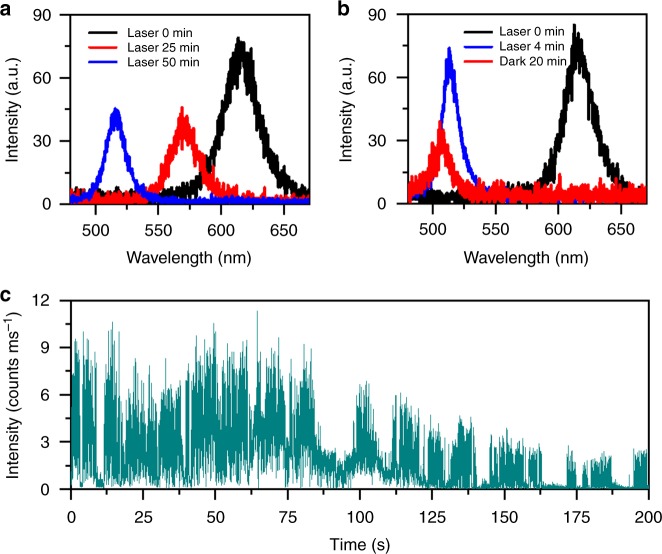
Light-induced phase segregation in single CsPbBr_1.2_I_1.8_ nanocrystals (NCs). **a** Photoluminescence (PL) spectra measured at three time points of 0, 25, and 50 min with an integration time of 1 s for a single CsPbBr_1.2_I_1.8_ NC continuously excited at a laser power density of 6 W cm^−2^. **b** PL spectra measured at two time points of 0 and 4 min with an integration time of 1 s for a single CsPbBr_1.2_I_1.8_ NC continuously excited at a laser power density of 6 W cm^−2^. Then after the excitation laser beam had been blocked for 20 min, a PL spectrum was taken with an integration time of 1 s for this single NC also excited at 6 W cm^−2^. **c** Time-dependent PL intensity measured between 20 and 220 s for the single CsPbBr_1.2_I_1.8_ NC whose PL spectra are plotted in **b** and continuously excited at 6 W cm^−2^. The binning time for acquiring each data point for the PL intensity is 100 ms

We next diluted the sample solution by 1000 times to make a low-density ensemble film of CsPbBr_1.2_I_1.8_ NCs, whose PL peak was blue-shifted to 512 nm after 20 min of laser excitation at 120 W cm^−2^ and stayed there almost permanently with only a slight recovery to the original position in the dark (Supplementary Figure 4). We further diluted the above sample solution to make a solid film containing spatially isolated single CsPbBr_1.2_I_1.8_ NCs, which could be verified from the second-order photon correlation measurement (Supplementary Figure 5). As shown in Fig. 2a for a representative single CsPbBr_1.2_I_1.8_ NC excited at 6 W cm^−2^, the initial 615 nm PL peak was blue-shifted to 570 and 515 nm after 25 and 50 min of continuous laser excitation, which never reverted back to the red side in the dark. This irreversible blue shift in the PL was observed in all of the single CsPbBr_1.2_I_1.8_ NCs studied in our experiment and, in some cases, a slight blue shift could be further resolved in the dark. As can be seen in Fig. 2b for a single CsPbBr_1.2_I_1.8_ NC excited at 6 Wcm^−2^, the PL peak was blue-shifted from 616 to 513 nm after 4 min of laser excitation and moved to 508 nm after the excitation laser beam had been blocked for 20 min in the dark.

The reversible PL shift could only occur in the high-density ensemble film due to ion migration among nearby CsPbBr_1.2_I_1.8_ NCs, which is reminiscent of the anion-exchange interaction between solution CsPbBr_3_ and CsPbI_3_ NCs to form the mixed-halide CsPbBr_*x*_I_3-*x*_ NCs^32^. As suggested previously for bulk MAPbI_3_ films^31^, the iodide ions would migrate away from the light-illuminated area to the immediately adjacent regions due to the Coulomb repulsion effect. Once the light illumination is removed, the stored iodide ions in the surroundings could be driven by the concentration gradient^31^ or the mixing entropy^21,33^ to fill the vacancies left in the original position. The above scenario can be similarly applied to the high-density CsPbBr_1.2_I_1.8_ NCs, so long as they can be treated as a bulk film divided into numerous nanoscale domains by the intervening surfaces. The situation is quite different for the low-density ensemble film and the isolated single CsPbBr_1.2_I_1.8_ NCs showing a monotonic and irreversible blue shift in the PL. This can be temporarily attributed to the reduction of surface iodide ions and the subsequent sublimation into a gaseous form^34,35^, which have been shown to be quite efficient in bulk films of the MAPbI_3_ perovskites especially under the influence of an electric field^36^. Within a high-density film of CsPbBr_1.2_I_1.8_ NCs, the iodide ions would be dominantly channeled away from the laser excitation area, but part of them might still suffer from the above dissipation process in some isolated NCs. This could be a likely origin for the incomplete recoveries of the PL peak and intensity shown in Fig. 1b, d for the high-density ensemble film of CsPbBr_1.2_I_1.8_ NCs in the dark. We performed energy-dispersive spectroscopy (EDS) measurements on a high-density film of ensemble CsPbBr_1.2_I_1.8_ NCs, whose PL peak was first blue-shifted to about 520 nm under high-power laser excitation and then moved back in the dark to a stable value of about 600 nm. EDS characterizations were applied on both laser-unirradiated and -irradiated areas to yield the I/Br stoichiometric ratios of about 1.46 and 1.28, respectively, thus confirming the loss of iodine element in the light-induced degradation process.

In bulk perovskite films, it has been firmly established that trap states on the surface and at the grain boundary can capture photo-excited charge carriers to create a local electric field capable of promoting ion migration^31,37,38^. When an electron–hole pair is photo-excited in a single colloidal NC, one of the charge carriers (e.g., the electron) can be trapped on the surface, leaving behind an extra hole in the core that couples with the next electron–hole pair to form a charged exciton^39,40^. The NC would become non-emissive due to nonradiative Auger recombination of the charged exciton, while the PL intensity can be resumed only after the surface electron returns back to or the extra hole also leaves the core^39,40^. This PL blinking effect was also observed here in single CsPbBr_1.2_I_1.8_ NCs, as can be seen in Fig. 2c for a representative one whose PL intensity switched randomly between the bright and dark time periods. For a single electron trapped on the CdSe NC surface, a local electric field from several to tens of volts per micrometer could be created^41^. It should be this electric field that breaks iodide bonds in CsPbBr_1.2_I_1.8_ NCs, possibly by creating a local strain in an inverse piezoelectric process, which has been theoretically proposed in molecular dynamics simulations on the bulk films of MAPbBr_*x*_I_3-*x*_ perovskites^11^. Recently, a compressive strain of 1% was reported for the bulk films of MAPbI_3_ and CsPbI_3_ perovskites biased under an electric field of 3.7 V µm^−1^ (ref. ^42^), thus lending support to the above proposal. It should be noted that the high-power laser excitation employed in our experiment could create multiple excitons in a single CsPbBr_1.2_I_1.8_ NC to trigger the exciton-exciton annihilation effect^43^. This kind of Auger-mediated process would greatly increase the charging possibility of a single NC^40^ to make it more vulnerable to the local electric field, which could explain the larger blue shift observed in the PL peak of ensemble CsPbBr_1.2_I_1.8_ NCs under higher-power laser excitation (Fig. 1a, c).

### Electrical biasing of high-density CsPbBr_1.2_I_1.8_ NCs

**Fig. 3:**
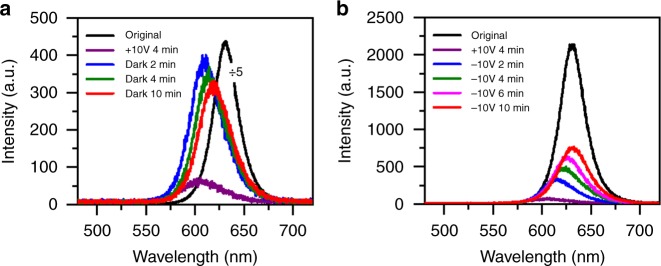
Phase segregation in ensemble film of CsPbBr_1.2_I_1.8_ nanocrystals (NCs) under electrical biasing. **a** Original photoluminescence (PL) spectrum measured for one position of the ensemble film without electrical biasing, together with the PL spectrum of this position measured after the ensemble film had been electrically biased for 4 min at 10 V in the dark. Also shown are three PL spectra of this position measured after the electrical biasing had then been removed for 2, 4, and 10 min. **b** Original PL spectrum measured for one position of the ensemble film without electrical biasing, together with the PL spectrum of this position measured after the ensemble film had been electrically biased for 4 min at 10 V in the dark. Also shown are four PL spectra of this position measured after a reverse electrical biasing of 10 V had been continuously supplied in the dark for 2, 4, 6, and 10 min. To acquire each of the PL spectra shown in **a** and **b**, the laser beam was unblocked for 1 s to excite the ensemble film with a power density of 6 W cm^−2^

To verify the influence of electric field on the phase segregation effect, we placed one solution drop of high-density CsPbBr_1.2_I_1.8_ NCs between two electrodes each with a width of 10 μm and separated by 5 μm in an interdigitated pattern. From bottom to top, the Au and Cr layers were deposited with the thicknesses of 80 and 5 nm, respectively. When an external electric field was applied between the two electrodes, there was no current flowing through the NC film so that electrical injection of charge carriers could be avoided. As shown in Fig. 3a for one position of the NC film, the PL intensity dropped significantly and the PL peak was blue-shifted from 631 to 607 nm after an electrical biasing of 10 V had been continuously supplied for 4 min in the dark. When this external electrical field was removed, the PL intensity recovered to about 20% of the initial value while the PL peak was red-shifted to reach 619 nm after the NC film had been left in the dark for 10 min (see Supplementary Figure 6 for a similar experiment performed on a low-density film of ensemble CsPbBr_1.2_I_1.8_ NCs where no red-shifted PL was observed after the external electric field had been removed). Consequently, the application of an electric field of 2 V µm^−1^ here to CsPbBr_1.2_I_1.8_ NCs could induce a similar PL shifting behavior to that from the laser excitation (see Fig. 1a, b). This is dramatically different from nearly all of the previous phase segregation studies on mixed-halide perovskites, wherein the excited-state charge carriers were exclusively required from either light illumination^10,12^ or electrical injection^17,18^. It was proposed that these charge carriers could couple with the soft lattice to create a polaron^7,44,45^, while the resulting strain would be sufficient to drive halide segregation for the nucleation of iodine-rich domains^11,15,46,47^. This polaron picture is obviously not applicable to the phase segregation effect observed here in our CsPbBr_1.2_I_1.8_ NCs, the function of whose photo-excited charge carriers should be the creation of a local electric field after being trapped on the surface, as can be deduced from the single-particle PL blinking phenomenon displayed in Fig. 2c.

Besides contributing to polaron formation, the excited-state charge carriers have also been deduced to drive the hopping of halide vacancies^48,49^ in the crystal lattice with an activation energy ranging from about 0.1 to 0.6 eV for mixed-halide perovskites^9,21,24,26,31,37,50^. In this sense, the external electric field applied by us to the high-density CsPbBr_1.2_I_1.8_ NCs might serve a similar role to energetically migrate iodide ions into their vacancies far away from the detection area. In this vacancy picture for an isolated single CsPbBr_1.2_I_1.8_ NC under laser excitation, the final destination for the migrated iodide ions could only be the surface vacancies where their Pb-I bond would be still conserved. It is then impossible for all of these iodide ions to sublime away and some of them should move back in the dark to fill the leftover vacancies in the core, which contradicts a further blue shift in the PL shown in Fig. 2b for a typical single CsPbBr_1.2_I_1.8_ NC. Now neglecting the polaron and vacancy origins for the phase segregation process, we can reasonably assume that the iodide ions should stay loosely on the surface or as interstitials in the CsPbBr_1.2_I_1.8_ NC lattice after the Pb-I bond is broken by the local or external electric field.

In Fig. 3b, we plot the PL spectra measured for one position of the high-density film of CsPbBr_1.2_I_1.8_ NCs formed between two other electrodes, which was also blue-shifted from 631 to 607 nm under an electrical biasing of 10 V for 4 min in the dark. When this electrical biasing was reversed to −10V and maintained for 10 min in the dark, the 607 nm PL peak could shift all the way back to the initial 631 nm, while an elongated biasing would shift this PL peak to the blue side again. Compared to natural recovery in the dark for the PL peak shown in Fig. 3a, the application of a reverse electric field obviously accelerated reflowing process of the freed iodide ions. For a specific single CsPbBr_1.2_I_1.8_ NC, the number of returning iodide ions should be larger than that of the escaping ones whose Pb-I bonds are broken again by the electric field, which can be invoked to explain the continuous red shift as well as the complete recovery of the PL peak observed in Fig. 3b.

### Other mechanisms for blue-shifted PL

Besides the migration of iodide ions to yield bromine-rich perovskite NCs, it is necessary to figure out whether there exist other mechanisms that can cause an enlarged energy bandgap with the blue-shifted PL. It is well known that the energy bandgap of all-inorganic perovskite NCs would increase with the increasing temperature^23^, however, this laser heating possibility can be safely ruled out since the blue-shifted PL was also induced by the application of an electric field (Fig. 3a). Meanwhile, the cooling process of CsPbBr_1.2_I_1.8_ NCs should not be different in the dark for a high-density ensemble film and an isolated single NC, but no returning of the PL to the red side was observed in the latter case (Fig. 2a, b). Moreover, we also excited ensemble CsPbBr_1.2_I_1.8_ NCs with a laser energy below their bandgap without seeing any blue-shifted PL (Supplementary Figure 7), thus again excluding possible contribution of the laser heating effect. Another possible mechanism for the variation of energy bandgap could be lattice distortion in CsPbBr_1.2_I_1.8_ NCs after the migration of iodide ions^12^. It was shown previously in bulk MAPbI_3_ perovskites^51–53^ that light illumination could alter the Pb-I bond angle and length, causing either lattice expansion or contraction that was able to decrease or increase the energy bandgap, respectively. To completely exclude the contribution of bromine enrichment to the enlarged energy bandgap, we synthesized single-halide perovskite CsPbI_3_ NCs (see Methods) to probe whether the migration of iodide ions and the subsequent lattice distortion could occur to induce a blue shift in the PL.

**Fig. 4:**
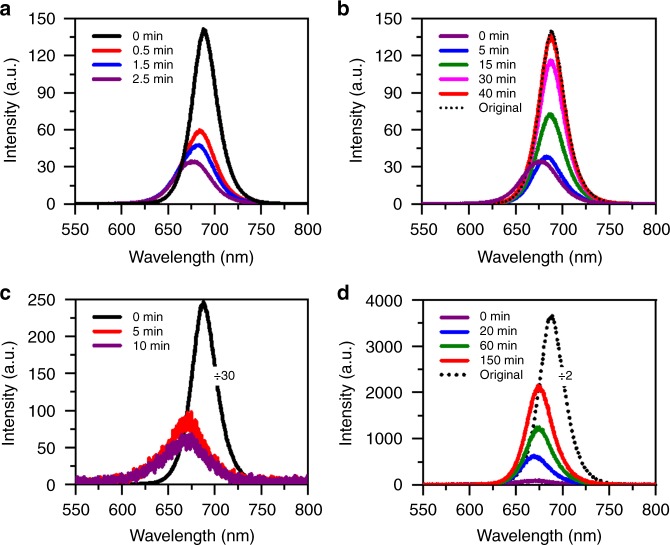
Light-induced migration of iodide ions in ensemble film of CsPbI_3_ nanocrystals (NCs). **a** Photoluminescence (PL) spectra measured at 0, 0.5, 1.5, and 2.5 min for one position of the ensemble film continuously excited at a laser power density of 15 kW cm^−2^. The PL spectrum at each time point was acquired with an integration time of 1 s. **b** PL spectra measured for the same position of the ensemble film after the excitation laser had then been blocked for 0, 5, 15, 30, and 40 min. At each time point, the laser beam was unblocked for 1 s to acquire the PL spectrum still at 15 kW cm^−2^. The original PL spectrum measured at the beginning of the laser excitation in **a** is also plotted. **c** PL spectra measured at 0, 5, and 10 min for another position of the ensemble film continuously excited at a laser power density of 15 kW cm^−2^. The PL spectrum at each time point was acquired with an integration time of 1 s. The PL spectrum measured at 0 min has been scaled down by 30 times for a good comparison. **d** PL spectra measured for this position of the ensemble film after the excitation laser had then been blocked for 0, 20, 60, and 150 min. At each time point, the laser beam was unblocked for 1 s to acquire the PL spectrum still at 15 kW cm^−2^. The original PL spectrum measured at the beginning of the laser excitation in **c** is also plotted but scaled down by two times

Compared to mixed-halide CsPbBr_1.2_I_1.8_ NCs whose light-induced phase segregation could be initiated at a laser power density smaller than 30 W cm^−2^ (Fig. 1a), the single-halide CsPbI_3_ NCs kept almost a stable PL peak under high-power laser excitation up to 10 kW cm^−2^. As shown in Fig. 4a for one position of the high-density film of ensemble CsPbI_3_ NCs, the PL peak was blue-shifted from the initial 689 to 678 nm after 2.5 min of laser excitation at 15 kW cm^−2^. It then shifted back in the dark to arrive at 688 nm after 40 min, with the original intensity being completely recovered (Fig. 4b). The blue shift observed here in the PL peak of ensemble CsPbI_3_ NCs, which could revert back to the red side in the dark, is different from that shown previously in single CsPbI_3_ NCs where layer-by-layer decomposition caused a permanent shrinkage of the NC size^54^. With elongated laser excitation still at 15 kW cm^−2^, the PL peak monitored for another position on the CsPbI_3_ NC film was blue-shifted from 688 to 668 nm after 10 min with a drastic intensity drop by about 100 times (Fig. 4c). When the NC film was left in the dark, the PL peak of the same position shifted to the red side and stabilized at 675 nm after 150 min, while the PL intensity recovered partially to about 30% of the original value (Fig. 4d). We further diluted the sample solution by 15 times to make a low-density film of ensemble CsPbI_3_ NCs, whose PL peak measured for one position was blue-shifted from 689 to 682 nm after 10 min of laser excitation at 15 kW cm^−2^ (Supplementary Figure 8). This PL peak still stayed at 682 nm even after the ensemble NC film had been left in the dark for 150 min, implying that the migration of iodide ions did occur among the high-density CsPbI_3_ NCs according to our previous discussions on the mixed-halide CsPbBr_1.2_I_1.8_ NCs (see Fig. 2a, b, as well as Supplementary Figure 4). The above optical measurements on the single-halide CsPbI_3_ NCs strongly suggest that, in addition to the dominant role played by bromine enrichment, a minor contribution of lattice distortion by the migration of iodide ion to the enlarged energy bandgap should not be neglected in the phase segregation process of CsPbBr_1.2_I_1.8_ NCs.

## Discussion

In conclusion, the phase-segregation effect is manifested in mixed-halide CsPbBr_1.2_I_1.8_ NCs as a blue shift in the PL peak upon laser excitation, which can return back to the red side in the dark for the high-density ensemble film but not the isolated single NCs. Since this blue-shifted PL can also be induced by electrical biasing of the ensemble film, we believe that the light-induced phase segregation process should be triggered by a local electric field generated after the photo-excited charge carriers are trapped on the NC surface. This local electric field would exert strain on the crystal lattice to cause bond breaking of the iodide ions, which then migrate away from the laser-excitation area in the high-density ensemble film or sublime into a gaseous form from the surface of isolated single CsPbBr_1.2_I_1.8_ NCs. Except the blue-shifted PL, we believe that the above discussions could also be employed to describe the phase segregation process observed in all the other mixed-halide perovskite materials.

It is well known that light-induced phase segregation in the bulk film of mixed-halide perovskites is universally causing a red shift in the PL peak due to the formation of both bromine- and iodine-rich domains, with the photo-excited excitons being trapped in the latter one with a smaller energy bandgap. The formation of both bromine- and iodine-rich domains in a single CsPbBr_1.2_I_1.8_ NC could be safely ruled out since a light-induced blue shift was observed in the PL peak. Meanwhile, recovery of this PL peak to the red side could be observed in the dark only for high-density films of ensemble CsPbBr_1.2_I_1.8_ NCs, thus strongly suggesting that the iodide ions do migrate among neighboring NCs. Since a spatial size of ~8 nm^11^ is normally needed for the formation of iodine-rich domain in the bulk films of mixed-halide perovskites, we speculate that this requirement might be difficult to be fulfilled in a single CsPbBr_1.2_I_1.8_ NC studied here with a lateral size of ~23 nm.

## Methods

### Cs-oleate precursor preparation

The Cs-oleate precursor was prepared by loading a mixture of 0.814 g Cs_2_CO_3_, 2.5 mL oleic acid (OA) and 30 mL octadecene (ODE) into a 100 mL three-neck flask. Under vacuum, the solution was dried and degassed for 10 min, and then heated at 120 °C for 1 h. The resulting solution was further heated to 150 °C under N_2_ gas and kept for 2 h until a clear solution was obtained. After being stored at room temperature, the Cs-oleate solution was always preheated to 120 °C before being used for the syntheses of CsPbBr_1.2_I_1.8_ and CsPbI_3_ NCs.

### Synthesis of mixed-halide CsPbBr_1.2_I_1.8_ NCs

0.105 g PbI_2_, 0.052 g PbBr_2_, 1.0 mL OA, 1.0 mL oleylamine (OAm) and 10 mL ODE were loaded into a 25 mL 3-neck flask. Under vacuum, the mixture solution in the flask was degassed for 10 min and then dried for 1 h at 120 °C. After the flask was connected to N_2_ gas, the solution temperature was raised to 160 °C and kept for 10 min, after which 1.0 mL of the Cs-oleate solution preheated to 120 °C was quickly injected and the reaction was stopped with ice bath after 5 s. The formed CsPbBr_1.2_I_1.8_ NCs were precipitated by adding tert-butanol and centrifuged for 20 min at 5000 rpm. The precipitate was redispersed in 2.0 mL hexane and centrifuged again for 20 min at 5000 rpm. Finally, the products were dispersed in 10 mL hexane or toluene and centrifuged for 10 min at 5000 rpm to remove any possible aggregates.

### Synthesis of single-halide CsPbI_3_ NCs

0.174 g PbI_2_, 1.0 mL OA, 1.0 mL OAm and 10 mL ODE were mixed in a 25-mL three-neck flask. After being degassed for 10 min, the mixture was heated to 120 °C and kept for 1 h under vacuum. The flask was then connected to N_2_ gas and the solution temperature was raised to 160 °C. 1.0 mL of the Cs-oleate solution preheated to 120 °C was quickly injected into the hot flask and the reaction was stopped with ice bath after 5 s. The resulting solution was centrifuged for 20 min at 5000 rpm, while the precipitated CsPbI_3_ NCs were dispersed in 2.0 mL hexane and centrifuged again for 20 min at 5000 rpm for further purification. Finally, the products were dispersed in 10 mL hexane or toluene and centrifuged for 10 min at 5000 rpm to remove any possible aggregates.

### Optical characterizations

The CsPbBr_1.2_I_1.8_ and CsPbI_3_ NC samples could be used directly after synthesis or stored for several months in a glove box filled with Ar gas, while very similar phase-segregation properties were observed from the fresh and stored samples. One drop of the concentrated or diluted solution of colloidal NCs (CsPbBr_1.2_I_1.8_ or CsPbI_3_) was spin-coated onto a fused silica substrate to form a solid film for the ensemble or single-particle optical characterizations at room temperature. The 405 nm output of a picosecond diode laser operated at a repetition rate of 5 MHz was focused onto the sample substrate by an immersion-oil objective (numerical aperture, 1.4). PL signal of the ensemble NCs or a single NC was collected by the same objective and sent through a 0.5 m spectrometer to a charge-coupled-device camera for the PL spectral measurement with an integration time of 1 s. The PL signal of a single NC can be alternatively sent through a non-polarizing 50/50 beam splitter to two avalanche photodiodes for the second-order photon autocorrelation measurement. When excited at the laser power densities specified in the text, the CsPbBr_1.2_I_1.8_ and CsPbI_3_ NCs always showed a decrease in the PL intensity and a blue shift in the PL peak at the beginning of the optical measurement, and no PL enhancement was observed from the photo-activation effect previously reported for semiconductor perovskite materials^55^.

## Supplementary information

Supplementary Information

## Data Availability

The data supporting the findings of this study are available from the corresponding authors upon request.
