# Supplementary material for: Phase segregation due to ion migration in all-inorganic mixed-halide perovskite nanocrystals
Source: Nat Commun. 2019 Mar 6;10:1088. doi: 10.1038/s41467-019-09047-7 (PMC6403211; doi:10.1038/s41467-019-09047-7)
Supplement: Supplementary file 1 — Supplementary Information [file 41467_2019_9047_MOESM1_ESM.pdf]

SUPPLEMENTARY INFORMATION

Phase Segregation Due to Ion Migration in All-Inorganic Mixed-Halide Perovskite Nanocrystals

Huichao Zhang[1,3†], Xu Fu[1†], Ying Tang[1], Hua Wang[2], Chunfeng Zhang[1], William W. Yu[2], Xiaoyong Wang[1*], Yu Zhang[2*], and Min Xiao[1,4*]

[1]*National Laboratory of Solid State Microstructures, School of Physics, and Collaborative Innovation Center of Advanced Microstructures, Nanjing University, Nanjing 210093, China*

[2]*State Key Laboratory of Integrated Optoelectronics and College of Electronic Science and Engineering, Jilin University, Changchun 130012, China*

[3]*College of Electronics and Information, Hangzhou Dianzi University, Xiasha Campus, Hangzhou 310018, China*

[4]*Department of Physics, University of Arkansas, Fayetteville, Arkansas 72701, USA*

[†]These authors contributed equally to this work.

[*]Correspondence to X.W. (wxiaoyong@nju.edu.cn), Y.Z. (yuzhang@jlu.edu.cn), or M.X. (mxiao@uark.edu).

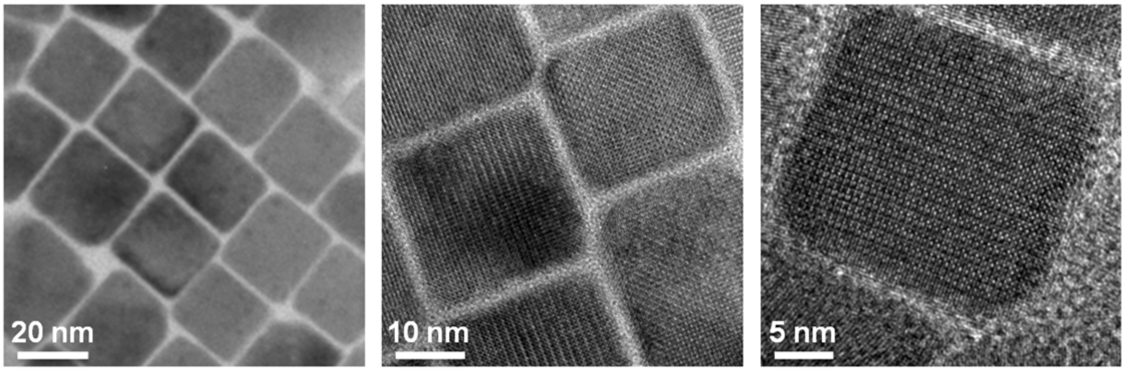

Supplementary Figure 1. Transmission electron microscopy (TEM) images of mixed-halide $CsPbBr_{1.2}I_{1.8}$ NCs.

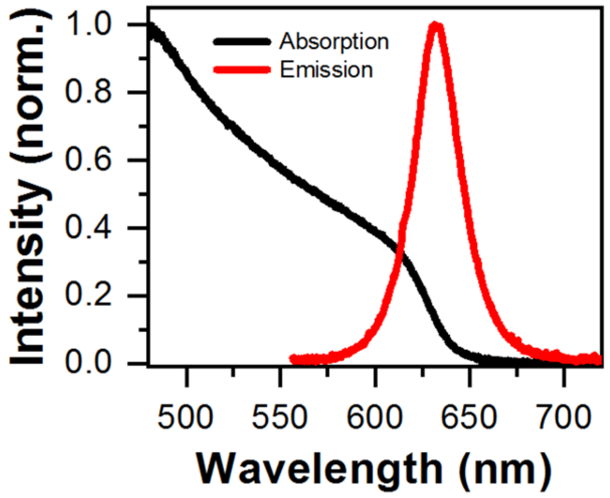

Supplementary Figure 2. Solution absorption and emission spectra measured at room temperature for mixed-halide $CsPbBr_{1.2}I_{1.8}$ NCs.

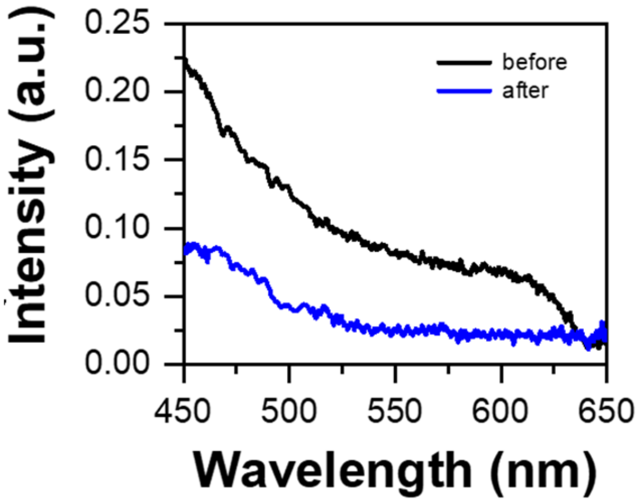

Supplementary Figure 3. Absorption spectra of ensemble $CsPbBr_{1.2}I_{1.8}$ NCs measured before and after 405 nm laser excitation with the following procedure. A high-density film of ensemble $CsPbBr_{1.2}I_{1.8}$ NCs was deposited on the fused silica substrate, while the two beams from the 405 nm laser and a white light source were focused on the sample center with a spot size of about 10 μm. With the 405 nm laser beam being blocked, the transmission spectrum of the white light beam was measured with an integration time of 1 s. Then the white light beam was blocked and the PL peak of ensemble $CsPbBr_{1.2}I_{1.8}$ NCs was blue-shifted to about 520 nm with continuous excitation of the 405 nm laser beam. With the 405 nm laser beam being blocked again, another transmission spectrum of the white light beam was measured. For reference, the white light spectrum was also measured after being transmitted through a blank fused silica substrate so that the above two absorption spectra of $CsPbBr_{1.2}I_{1.8}$ NCs could be normalized.

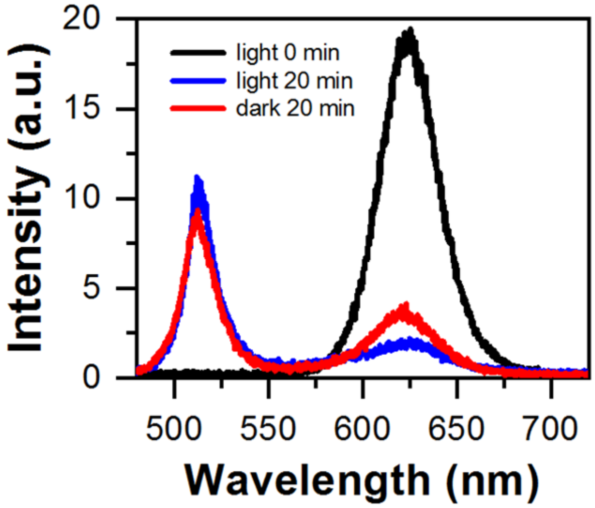

Supplementary Figure 4. PL spectra measured at two time points of 0 and 20 min with an integration time of 1 s for a low-density ensemble film of $CsPbBr_{1.2}I_{1.8}$ NCs continuously excited at a laser power density of 120 W cm^{-2}. Then after the excitation laser beam had been blocked for 20 min, a PL spectrum was taken with an integration time of 1s for this ensemble film also excited at 120 W cm^{-2}.

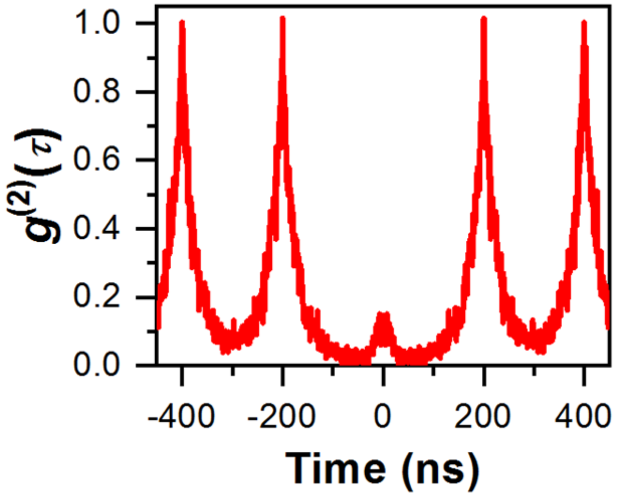

Supplementary Figure 5. Second-order photon correlation function of $g^{(2)}(\tau)$ measured for a single $CsPbBr_{1.2}I_{1.8}$ NC, which gives the probability of detecting two consecutively-emitted photons at variable time delays of τ. If an excitation pulse has already triggered a photon from a single NC, no other photons would be emitted within the pulse duration to result in a missing peak or a vanishing $g^{(2)}(\tau)$ probability at zero time delay. The probability of registering a second photon would be high with the arrivals of other excitation pulses, so that one could observe all those peaks at the integer multiples of the pulse separation of ~200 ns. The normalized $g^{(2)}(\tau)$ value of 0.15 measured at $\tau = 0$ unambiguously confirms that a single $CsPbBr_{1.2}I_{1.8}$ NC is excited with an extremely small probability of emitting more than one photon at a time.

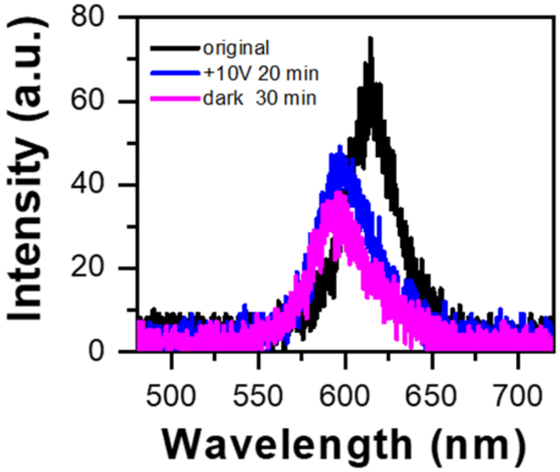

Supplementary Figure 6. Original PL spectrum measured for one position on a low-density film of ensemble $CsPbBr_{1.2}I_{1.8}$ NCs without electrical biasing, together with the PL spectrum of this position measured after the ensemble film had been electrically biased for 20 min at 10 V in the dark. Also shown are the PL spectrum of this position measured after the electrical biasing had then been removed for 30 min. To acquire each PL spectrum, the laser beam was unblocked for 1 s to excite the ensemble film with a power density of 6 W cm^{-2}.

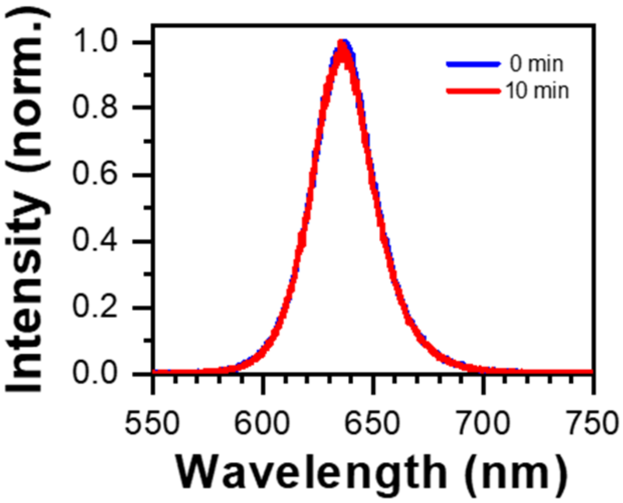

Supplementary Figure 7. PL spectra measured at 0 and 10 min for one film position of the ensemble $CsPbBr_{1.2}I_{1.8}$ NCs continuously excited with a 76 MHz picosecond 800 nm laser at a power density of 30 W cm^{-2}. PL spectrum at each time point was acquired with an integration time of 1 s using a 5 MHz picosecond 405 nm laser at a power density of 30 W cm^{-2}.

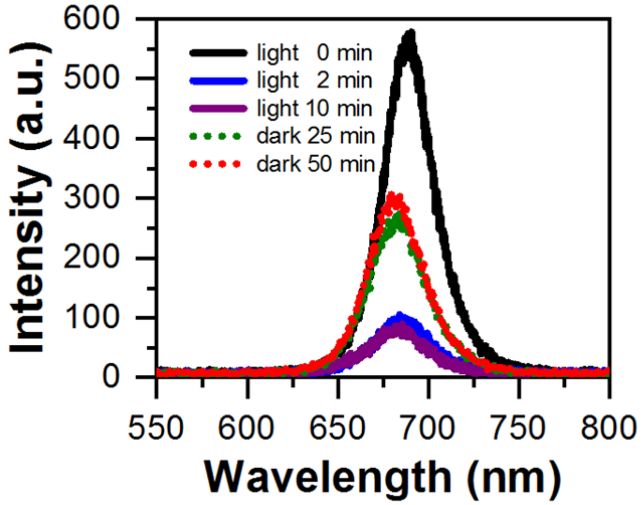

Supplementary Figure 8. PL spectra measured at 0, 2 and 10 min with an integration time of 1 s for one position of the low-density ensemble film of $CsPbI_3$ NCs continuously excited at a laser power density of 15 kW cm^{-2}. PL spectra measured for the same position of the ensemble film after the excitation laser beam had then been blocked for 25 and 50 min are also presented. At each time point, the laser beam was unblocked for 1 s to acquire the PL spectrum still at 15 kW cm^{-2}.
